# Evolutionary perspectives on endometrial cancer: antagonistic pleiotropy

**DOI:** 10.1080/23723556.2026.2681248

**Published:** 2026-06-11

**Authors:** Hiroshi Kobayashi

**Affiliations:** a Department of Obstetrics and Gynecology, Nara Medical University, Kashihara, Japan; b Department of Gynecology and Reproductive Medicine, Ms.Clinic MayOne, Kashihara, Japan

**Keywords:** Antagonistic pleiotropy, endometrial cancer, evolutionary trade-off, p53, PI3K

## Abstract

The incidence of endometrial cancer is increasing worldwide, with a particularly marked rise in early-onset cases. Modern environmental and lifestyle factors, including low parity, delayed childbirth, obesity, insulin resistance, and chronic inflammation, are thought to contribute to this trend. Although TCGA-based molecular classification has revealed substantial heterogeneity—such as defects in DNA repair, constitutive activation of the PI3K–AKT–mTOR pathway, and disruption of the p53 pathway—the reasons these abnormalities preferentially accumulate in endometrial cancer remain incompletely understood. In this review, we reinterpret the molecular pathogenesis of endometrial cancer through the frameworks of evolutionary mismatch and antagonistic pleiotropy, conceptualizing the disease as a continuum linking reproduction, aging, and tumorigenesis. We integrate evidence from molecular oncology, reproductive biology, and evolutionary medicine to examine how key signaling pathways acquire dual roles across the lifespan. The PI3K–AKT–mTOR pathway is indispensable for reproductive function and endometrial regeneration, yet becomes chronically overactivated under contemporary conditions of overnutrition and obesity, thereby promoting hyperplasia and tumorigenesis. Similarly, the p53 pathway supports genome integrity and placental function during reproduction, but age- and inflammation-associated dysregulation induces cellular senescence and facilitates tumor progression. Importantly, these pathways interact and reinforce each other, amplifying oncogenic phenotypes such as TP53 mutations and PI3K hyperactivation. Collectively, the molecular alterations observed in endometrial cancer can be understood as maladaptive consequences of reproductive systems shaped by evolution but exposed to modern environments. Therefore, an evolutionary medicine perspective may provide a coherent framework for understanding the development of endometrial cancer.

## 1. Introduction

Endometrial cancer is the most common gynecologic malignancy, and in recent years a global increase in incidence, together with a downward shift in age at onset, has been reported.^1^ These trends are thought to reflect the combined effects of changes in modern environments and reproductive behaviors.^1^ In particular, alterations in reproductive patterns—such as reduced parity, increasing age at first childbirth, and shorter durations of breastfeeding—have prolonged the lifetime exposure of women to anovulatory cycles and unopposed estrogen, thereby increasing the risk of hormone-dependent tumors.^2^ In addition, environmental and metabolic mismatches associated with sedentary lifestyles, endocrine disruption by environmental chemicals, and increased detection of endometrial hyperplasia due to improved access to medical care are also considered contributors to the rising incidence.^3^ Furthermore, estrogen excess and chronic inflammation accompanying the growing prevalence of obesity and insulin resistance strongly promote endometrial proliferation and tumorigenesis.^4^ Through the interaction of these factors, the incidence of endometrial cancer at younger ages has increased compared with previous decades.^1^

Clinically and molecularly, endometrial cancer is highly heterogeneous. Traditionally, it has been classified into type I (estrogen-dependent) and type II (estrogen-independent) tumors; however, this dichotomous model has proven insufficient to fully explain the underlying molecular pathophysiology.^5^ Comprehensive analyzes by The Cancer Genome Atlas (TCGA) reclassified endometrial cancer into four groups—POLE ultramutated (POLEmut), microsatellite instability–high (MSI), copy-number low (CNL/NSMP), and copy-number high/p53-abnormal (CNH)—and revealed a multilayered molecular landscape centered on defects in DNA repair, dysregulation of the PI3K–AKT–mTOR pathway, and disruption of the p53 pathway.^6^ Nevertheless, why these molecular abnormalities preferentially accumulate in endometrial cancer, and why the incidence of this disease is increasing in modern societies, cannot be fully explained by molecular classification alone.

Recently, increasing attention has been directed toward reinterpretation of these molecular abnormalities from an evolutionary biological perspective. In particular, the concepts of evolutionary mismatch^7^ and antagonistic pleiotropy (AP) theory^8^ ^,^ ^9^ provide useful frameworks for understanding the relationship between reproductive function, aging, and tumorigenesis. Humans evolved under past environments typified by low energy availability, high physical activity, and high fertility, as seen in hunter–gatherer societies. In contrast, modern societies expose individuals to conditions that were not anticipated during evolution, including high-calorie diets, chronic overnutrition, reduced physical activity, and low parity with delayed childbirth. The evolutionary mismatch framework posits that this discordance increases the risk of many chronic diseases, such as obesity, type 2 diabetes, and cardiovascular disease.^10^ ^,^ ^11^ AP theory describes a “trade-off between benefits and costs,” whereby certain genes or signaling pathways confer advantages for reproduction and survival early in life but exert deleterious effects later in life, such as promotion of aging and tumorigenesis. As a consequence of natural selection, such late-life pathological effects are less likely to be eliminated.^9^

In this review, we aim to integrate the molecular pathogenesis of endometrial cancer as a continuum linking reproductive function, aging, and tumorigenesis by introducing the evolutionary medicine concepts of antagonistic pleiotropy and evolutionary mismatch. Specifically, after outlining both the TCGA-based and classical classifications, we reinterpret the PI3K–AKT–mTOR and p53 pathways within an evolutionary biological framework. We further discuss how the appropriate activity levels and dysregulation of the p53–mTOR–senescence response network, positioned at the interface of decidualization, cellular senescence, and tumorigenesis in the endometrium, correspond to the mechanisms underlying the development of endometrial cancer.

## 2. Materials and methods

### 2.1. Search strategy and selection criteria

This review was conducted as a narrative review with the aim of integrating molecular classifications of endometrial cancer, major signaling pathways that support fundamental life processes, antagonistic pleiotropy (AP), evolutionary mismatch, and perspectives from life history theory (Life History Theory). Literature searches were performed using PubMed and Google Scholar, covering publications up to October 2025. Search terms were used in various combinations and included “endometrial cancer,” “TCGA classification,” “hormonal signaling,” “PI3K,” “AKT,” “mTOR,” “AMPK,” “p53,” “cellular senescence,” “evolutionary mismatch,” “antagonistic pleiotropy,” “life history theory,” “fertility,” and “reproductive aging.”

The literature considered comprised original research articles, review articles, meta-analyzes, studies in genetics and population genetics, and seminal papers in evolutionary medicine. High-impact publications in the fields of gynecologic oncology, molecular biology, aging and regenerative biology, evolutionary biology, and comparative reproductive biology were preferentially referenced. Established conceptual frameworks—including the TCGA classification of endometrial cancer, reproductive strategies based on life history theory, the evolutionary mismatch hypothesis, and antagonistic pleiotropy theory—were adopted as the basis for interpretation. Using these frameworks, we systematically organized how each concept relates to the molecular pathophysiology and age-associated characteristics of endometrial cancer.

Literature selection focused primarily on the following categories: (i) studies addressing the molecular classification of endometrial cancer, particularly TCGA subtypes; (ii) studies examining p53 signaling and the PI3K–AKT–mTOR pathway; (iii) studies investigating evolutionary trade-offs between reproduction and cancer; and (iv) studies on human reproductive strategies and disease risk grounded in life history theory. By integrating evidence across these multiple disciplines, we reconstructed the risk of endometrial carcinogenesis from a multifaceted perspective encompassing aging, reproduction, metabolism, and evolution, and developed the discussion presented in this review.

## 3. Results

### 3.1. Classification of endometrial cancer

Comprehensive genomic analyzes by The Cancer Genome Atlas (TCGA) established a framework that classifies endometrial cancer into four molecular subtypes: POLE ultramutated (POLEmut), microsatellite instability–high (MSI), copy-number low (CNL), and copy-number high (CNH).^6^ The POLEmut subtype is characterized by exonuclease domain mutations in POLE (e.g., P286R, V411L), resulting in an extremely high mutational burden. This leads to robust T-cell–mediated immune responses driven by neoantigen generation and is associated with an exceptionally favorable prognosis. Although these tumors harbor diverse subclones, they exhibit convergent evolution in pathways such as PI3K signaling.^12^ Indeed, mutations in PI3K–AKT–mTOR pathway–related genes are detected at high frequencies, including PTEN (94%), PIK3CA (71%), and PIK3R1 (65%).^13^ Even when histologically high grade, the risk of recurrence is low, providing a rationale for postoperative treatment de-escalation.

The MSI subtype is primarily based on mismatch repair (MMR) deficiency due to hypermethylation of the MLH1 promoter and accounts for approximately 77% of MSI cases. MLH1 silencing is associated with epigenetic alterations as well as aging and inflammation, and this subtype is characterized by high intratumoral genetic heterogeneity.^12^ Aberrations in the PI3K pathway are detected in approximately 95% of cases.^13^ Owing to its high mutational burden and prominent immune cell infiltration, this subtype shows high sensitivity to immune checkpoint inhibitors.

The CNL subtype predominantly consists of low- to intermediate-grade endometrioid carcinomas, is estrogen receptor (ER)– and progesterone receptor (PR)–positive, and is associated with a favorable prognosis. In more than 90% of cases, clonal accumulation of mutations in the PI3K–AKT–mTOR pathway—centered on PTEN, PIK3CA, and PIK3R1—drives a pattern of linear tumor evolution.^12^ Mutations in KRAS, CTNNB1, and ArId1A frequently coexist, and interactions with the cyclic proliferative environment of the endometrium are thought to contribute to tumorigenesis.

The CNH subtype represents a high-grade malignancy characterized by obligatory TP53 mutations, marked genomic instability, and high proliferative capacity, and is associated with poor prognosis. Under conditions of pronounced chromosomal instability, cells harboring p53 abnormalities are preferentially selected, corresponding to the classical type II category.

Clinically, the ESMO 2022 guidelines recommend a molecular diagnostic algorithm in which POLE mutations, MMR deficiency, and p53 abnormalities are assessed sequentially, with tumors that do not meet these criteria classified as no specific molecular profile (NSMP). This approach has facilitated the integration of TCGA classification into routine clinical practice.^14^ Among low- to intermediate-grade tumors, the relative frequencies are approximately CNL (63%), MSI (25%), POLEmut (6%), and CNH (5%), whereas in grade 3 tumors, the proportions of MSI, POLEmut, and CNH increase. Notably, grade 3 tumors comprise a mixture of prognostically favorable POLEmut cases and poor-prognosis p53-abnormal cases; therefore, morphological assessment alone is insufficient, and molecular classification is essential. In Asian populations, including Japan, the prevalence of the POLEmut subtype is reported to be lower than in Western countries, whereas MSI and CNH subtypes appear somewhat more frequent. However, given differences in patient backgrounds and analytical methodologies, large-scale studies are required to clarify these observations.^15-17^

#### 3.1.1. Mechanisms of endometrial carcinogenesis

Endometrial cancer arises through multistep and multifactorial processes rather than from a single causative factor. Hormonal and metabolic environments, such as estrogen excess and obesity-associated hyperinsulinemia, interact with genetic alterations—including point mutations, copy number variations, and epigenetic changes—to drive malignant transformation.^6^ Type I endometrial cancer comprises estrogen-dependent endometrioid tumors and is largely composed of the POLEmut, MSI, and CNL molecular subtypes. Its molecular basis can be summarized along two principal axes: preservation of wild-type p53 and frequent disruption of the PI3K–AKT–mTOR pathway.^6^ Loss of PTEN function and activating mutations in PIK3CA constitutively activate this pathway, thereby promoting hormone-dependent proliferation and cell-cycle progression.^18^ ^,^ ^19^ The sensitivity of tumors harboring these alterations to PI3K or mTOR inhibitors supports the validity of this pathway as a therapeutic target.^20^ In addition, KRAS mutations enhance proliferative capacity via the Ras–MAPK cascade, while CTNNB1 mutations aberrantly activate the Wnt/β-catenin pathway, characterizing a subset of low-grade tumors by nuclear accumulation of *β*-catenin.^6^ Mutations in the chromatin remodeling factor ArId1A profoundly alter chromatin architecture and transcriptional programs. In the POLEmut subtype, despite an extremely high mutational burden, p53 function is preserved, resulting in strong antitumor immune responses and favorable clinical outcomes. Furthermore, the evolutionary patterns of PI3K pathway mutations differ among subtypes: in NSMP tumors, mutations tend to accumulate early and uniformly, whereas in MMR-deficient and POLEmut tumors, such mutations may arise later or independently while maintaining substantial intratumoral heterogeneity.^12^

In contrast, type II tumors are characterized by chromosomal instability and widespread copy number alterations, with TP53 mutations at their core. Loss of p53 function leads to genomic instability, dysregulation of cell-cycle control, and evasion of apoptosis, directly underpinning the aggressive clinical behavior of high-grade malignancies such as serous carcinoma.^6^ Moreover, mutations in FBXW7 and PPP2R1A, as well as amplification and activation of ERBB2, are frequently observed in serous carcinoma and contribute to increased malignancy by enhancing invasiveness and metastatic potential.^6^ Thus, specific combinations of genetic abnormalities largely determine the biological characteristics and prognosis of endometrial cancer, underscoring the essential role of molecular classification as a foundation for clinical decision-making.

### 3.2. Signaling pathways supporting fundamental life processes

**Figure 1.**
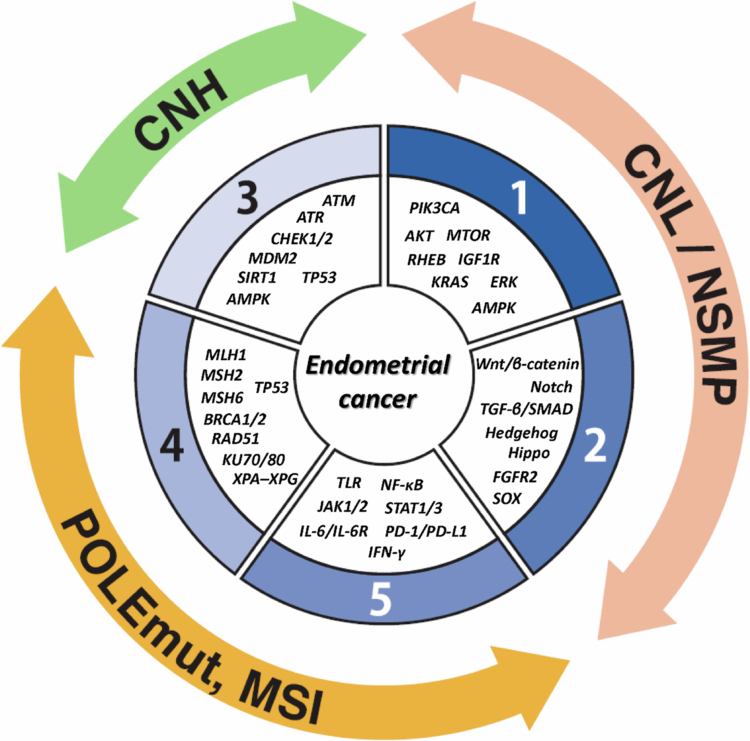
Integrated model of the five fundamental life domains and TCGA classification in endometrial cancer. This figure conceptualizes endometrial carcinogenesis by organizing it into five fundamental life domains: (1) growth and metabolic regulation, (2) cell fate determination and development, (3) genomic stability and stress responses, (4) DNA repair, and (5) immune responses. These domains are hierarchically integrated and collectively determine tumor development. Each domain is composed of representative signaling pathways, including PI3K–AKT–mTOR; Wnt/Notch/TGF-β; p53–ATM/ATR; mismatch repair (MMR) and homologous recombination (HR); and TLR/NF-κB/JAK signaling, which engage in extensive crosstalk to support cellular stability and adaptability. The figure further aligns the four TCGA molecular subtypes with these domains, illustrating that POLEmut and MSI tumors are primarily characterized by abnormalities in the DNA repair and immune domains, CNL tumors by dysregulation of growth, metabolic, and developmental control, and CNH tumors by breakdown of the genomic stability domain.

The signaling networks that sustain life are exceedingly complex, orchestrating cell proliferation, differentiation, metabolism, and the maintenance of homeostasis with remarkable precision. With regard to the biological characteristics of cancer, Douglas Hanahan and Robert A. Weinberg proposed the concept of the “Hallmarks of Cancer,” systematizing ten fundamental features common to tumor initiation and progression.^21^ According to their framework, cancer cells sustain proliferative signaling and evade growth suppressors. They also resist cell death and acquire replicative immortality. Furthermore, they induce angiogenesis to support tumor growth and activate invasion and metastasis to disseminate to distant sites. In addition, cancer cells reprogram energy metabolism and evade immune surveillance. These hallmarks are supported by enabling characteristics—namely genomic instability and mutation, as well as tumor-promoting inflammation—which together provide a comprehensive theoretical framework for understanding cancer development and progression. In the present review, these ten factors are reorganized into five core domains: (1) growth and metabolic regulation, (2) cell fate determination and development, (3) genome stability and stress responses, (4) DNA repair, and (5) immune responses^21-24^ (Figure 1). This systematic framework enables the conceptual and integrative organization of the molecular foundations underlying the development of endometrial cancer by recognizing at least five principal domains.

These domains function as partially independent modules that are hierarchically and dynamically integrated to form networks supporting organismal homeostasis and environmental adaptation. The growth and metabolic domain governs resource allocation and proliferation; the developmental and cell fate domain regulates differentiation and tissue homeostasis; the genomic stability and stress response domain enables rapid responses to cellular damage; DNA repair maintains the integrity of genetic information; and the immune response domain defends against external threats. Through their interconnections and context-dependent prioritization and regulation, these domains collectively generate the robustness and plasticity of living systems.

Within the growth and metabolic regulation domain, key components such as PIK3CA, AKT, MTOR, IGF1R, KRAS, ERK, and AMPK coordinate proliferative signals through the PI3K–AKT–mTOR, IGF/insulin, RAS–MAPK, and AMPK pathways, integrating mitogenic inputs with protein synthesis and metabolic activation.^25-28^ Although p53 is not a core constituent of this domain, it indirectly influences metabolic regulation, for example through suppression of mTOR activity.^29^ The cell fate determination and developmental domain includes pathways and factors such as Wnt/β-catenin, Notch, TGF-β/SMAD, Hedgehog, Hippo, FGFR2, and SOX family transcription factors, which collectively govern differentiation and tissue homeostasis.^30^ While p53 and PI3K–AKT–mTOR are not primary drivers in this domain, they maintain functional connections through extensive crosstalk with developmental signaling pathways.^30^ ^,^ ^31^ In the genomic stability and stress response domain, p53 serves as a central hub, forming a network with ATM, ATR, CHEK1/2, MDM2, SIRT1, and AMPK to regulate cell-cycle arrest, DNA repair, and apoptosis in response to DNA damage, metabolic stress, and oxidative stress.^32^ ^,^ ^33^ p53 functions as a key integrator of genome maintenance, whereas the PI3K–AKT–mTOR pathway participates largely as a regulated target that is suppressed through interactions with AMPK.^34^ The DNA repair domain encompasses major repair pathways, including mismatch repair, homologous recombination, non-homologous end joining, and nucleotide excision repair.^32^ In this context, p53 coordinates DNA repair processes through ATM/ATR-mediated signaling, whereas the PI3K–AKT pathway is not a direct structural component of DNA repair machinery.^35^ Within the immune response domain, signaling systems such as Toll-like receptors (TLRs), NF-κB, JAK–STAT, IL-6/IL-6R, PD-1/PD-L1, and IFN-*γ* regulate innate and adaptive immunity and contribute to inflammation and antitumor immune responses.^36^ ^,^ ^37^ p53 participates in immune regulation, for example by suppressing PD-L1 expression, but is not a central driver of this domain. Similarly, although the PI3K–AKT–mTOR pathway plays important roles in immune cell metabolism and differentiation, it does not constitute the primary axis of immune signaling.

#### 3.2.1. TCGA classification of endometrial cancer interpreted as disruption of life-function networks

The TCGA classification of endometrial cancer extends beyond a simple four-tier system based on genomic mutation patterns; its pathobiological significance becomes clearer when each subtype is interpreted in relation to the five functional domains that support fundamental life processes. In the POLEmut subtype, disruption of the DNA repair domain—particularly defects in the replication proofreading machinery—leads to an extremely high mutation rate. This, in turn, elicits a robust antitumor immune response and confers an excellent prognosis, highlighting the close interconnection between the DNA repair and immune domains. The MSI subtype is likewise grounded in DNA repair defects caused by mismatch repair (MMR) deficiency, while simultaneously exhibiting immune activation driven by increased neoantigen load, thereby constituting a dual-layered phenotype. In contrast, the CNL/NSMP subtype lacks overt DNA repair defects or p53 mutations and is characterized by dysregulation within the growth and metabolic regulation domain and the cell fate determination domain, exemplified by activation of the PI3K–AKT–mTOR pathway and abnormalities in hormonal responsiveness. The CNH subtype, on the other hand, is driven by loss of function of p53, a core component of the genomic stability and stress response domain, resulting in widespread copy number alterations and the emergence of highly aggressive phenotypes.

Thus, mapping the four TCGA subtypes onto the five life-function domains makes it possible to visualize which “foundational domain of life” is predominantly compromised in each subtype (Figure 1). Specifically, POLEmut and MSI tumors can be understood as disorders of the DNA repair–immune network; CNL tumors as cancers arising from dysregulation of growth, metabolism, and developmental control; and CNH tumors as manifestations of collapse of the genomic stability system itself. The TCGA classification therefore represents more than a set of genomic labels—it serves as an integrative indicator of which life domains have failed, substantially deepening our understanding of mechanisms of carcinogenesis, tumor biology, and therapeutic vulnerabilities.

#### 3.2.2. Universality and molecular diversity of signaling pathway dysregulation in endometrial cancer

Comprehensive analyzes by TCGA have demonstrated that in the major endometrial cancer subtypes—POLEmut, MSI, and CNL/NSMP—mutations in genes related to the PI3K–AKT–mTOR pathway, such as PTEN, PIK3CA, and PIK3R1, are observed at high frequency. In particular, single-cell analyzes of NSMP tumors have shown that nearly all tumor cells harbor one of these alterations, suggesting that PI3K pathway dysregulation constitutes a principal driver of tumorigenesis in this subtype.^12^ Moreover, even in the POLEmut subtype, PTEN and PIK3CA mutations are frequently present independently of the extreme mutational burden, indicating that PI3K–AKT–mTOR pathway abnormalities are widely shared as a “common background” rather than being restricted to specific subtypes.^13^

In contrast, the CNH subtype is characterized by an almost obligatory presence of TP53 mutations, and tumor development is driven by profound chromosomal instability resulting from disruption of genomic stability and stress response mechanisms. In this subtype, loss of the p53 pathway is considered the central determinant of tumor phenotype. Thus, although differences in mutational spectra exist among subtypes, two major axes—dysregulation of the PI3K–AKT–mTOR pathway and disruption of the p53 pathway—emerge as the principal molecular foundations supporting endometrial carcinogenesis. How these abnormalities are linked to aging and changes in lifestyle and environmental conditions to promote tumor development is discussed in the following section.

### 3.3. Physiological roles of signaling pathways in the endometrium and their conversion to tumorigenesis

#### 3.3.1. Physiological roles of the PI3K–AKT–mTOR pathway and its conversion to tumorigenesis

**Figure 2.**
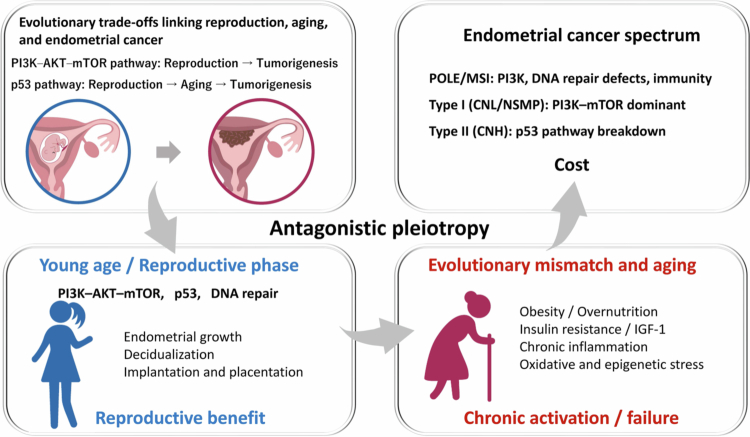
Developmental basis of endometrial cancer viewed through the lens of antagonistic pleiotropy. The upper left panel illustrates the physiological roles of the PI3K–AKT–mTOR pathway and p53 in the endometrium and their age-associated transition toward tumorigenesis. During the reproductive period, the PI3K–AKT–mTOR pathway and p53 contribute to successful pregnancy by supporting endometrial proliferation, regeneration, and the maintenance of genomic stability. In contrast, when regulation of these pathways becomes disrupted by aging or chronic stress, excessive proliferation, cellular senescence, and genomic instability progress, providing a foundation for tumorigenesis. The high regenerative capacity, hormone responsiveness, and growth and metabolic signaling of the endometrium are advantageous for maintaining fertility during the reproductive years (lower left); however, through interactions with age-related declines in DNA repair capacity, cellular senescence, chronic inflammation, and loss of regulatory control, these same features are converted into increased risks of abnormal proliferation and tumor development (lower right). The molecular mechanisms exemplified by p53 and the PI3K–AMPK–mTOR axis can thus be understood within the framework of antagonistic pleiotropy (AP), in which benefits in early life are accompanied by costs in later life (upper right).

The endometrium is a tissue that undergoes repeated cycles of proliferation and regeneration throughout the reproductive years in response to hormonal stimulation, during which growth and metabolic signaling centered on the PI3K–AKT–mTOR pathway plays a pivotal role (Figure 2, upper left). In early life, activation of this pathway by estrogen and IGF-1 contributes to the maintenance of ovarian function, endometrial regeneration, establishment of a receptive implantation environment, and successful pregnancy, thereby conferring adaptive benefits for reproduction.^38^ ^,^ ^39^ Accordingly, pathway activation driven by abnormalities in PTEN or PIK3CA may have been advantageous for tissue maintenance and regeneration during the reproductive period. However, with aging, cumulative burdens—including inflammation, oxidative stress, DNA replication stress, metabolic disturbances (obesity and hyperinsulinemia), and estrogen excess—convert constitutive activation of the PI3K–AKT pathway into a driver of genomic instability, hyperproliferation, evasion of apoptosis, and dysregulation of senescence control, thereby creating a foundation for tumorigenesis.

The PI3K–AKT pathway broadly regulates metabolism, proliferation, survival, cell-cycle progression, and transcriptional programs, influencing a wide range of downstream effectors such as mTOR, NF-κB, FOXO, GSK3β, and caspase-9.^40^ In particular, excessive mTOR activity can induce depletion of the ovarian reserve,^41^ premature ovarian aging, and endometrial hyperplasia.^42^ ^,^ ^43^ Moreover, hyperactivation of the mTOR pathway can promote cellular senescence.^44^ ^,^ ^45^ While senescence is beneficial as a tumor-suppressive mechanism during early and midlife, in later life the accumulation of senescent cells and their senescence-associated secretory phenotype (SASP) drives chronic inflammation and tissue dysfunction. Because the endometrium is highly sensitive to growth signals due to its lifelong cycles of proliferation and shedding, this intrinsic property may facilitate early clonal selection of cells harboring PTEN loss or PIK3CA mutations, thereby accounting for the high prevalence of PI3K pathway abnormalities in endometrioid endometrial cancer. Such clones are prone to acquire subsequent alterations, including Wnt pathway dysregulation, chromatin remodeling defects, and DNA repair abnormalities, consistent with a multistep model of carcinogenesis. Cells with PTEN loss or PIK3CA mutations thus exhibit proliferative advantages in early life but, under age-associated hormonal and metabolic environments, are redirected toward malignant transformation.

#### 3.3.2. Physiological roles of p53 and its transition toward tumorigenesis

p53 is a prototypical pleiotropic gene with multifaceted functions, including DNA damage responses, cell cycle regulation, apoptosis, senescence, SASP production, and metabolic regulation,^46-48^ and it is tightly interconnected with mTOR, AMPK, and autophagy pathways (Figure 2, upper left). During early life, p53 contributes to survival and reproduction through genome protection and tumor suppression, whereas its sustained activation under conditions of aging or chronic stress leads to stem cell dysfunction, impaired tissue regeneration, and acceleration of aging.^46^ ^,^ ^49^ This duality—benefits in youth and costs in old age—represents a canonical example of antagonistic pleiotropy (AP) embodied by p53.^46^ ^,^ ^50-58^

p53 also plays critical roles in reproduction. Reduced p53 activity increases the risk of preterm birth through premature senescence of the decidua and fetal membranes,^59^ ^,^ ^60^ whereas excessive p53 activation suppresses trophoblast proliferation and invasion and promotes apoptosis, thereby contributing to miscarriage and placental dysfunction.^61-63^ Accordingly, maintenance of an appropriate level of p53 activity is essential for the establishment and maintenance of pregnancy, and p53 function during the reproductive period itself exhibits AP-like properties.

p53 activity is finely regulated by ubiquitination centered on MDM2, as well as by post-translational modifications such as phosphorylation and acetylation. However, age-associated DNA damage, oxidative stress, disruption of stem cell niches, and epigenetic alterations modify its basal activity and responsiveness.^49^ ^,^ ^64^ ^,^ ^65^ Reduced p53 activity increases cancer risk due to impaired elimination of damaged cells, whereas excessive activity induces stem cell exhaustion and tissue atrophy, underscoring the importance of maintaining optimal p53 activity throughout life. Notably, the rs3820282 variant allele that enhances Wnt4 expression confers advantages for pregnancy and implantation but is associated with increased long-term risks of endometrial cancer and endometriosis,^66^ illustrating an evolutionary trade-off between reproductive benefit and late-onset disease risk.

The biological outcomes of p53 depend not on its mere presence or absence but on its dosage, duration of activation, mode of activation (acute/pulsatile versus chronic/sustained), and the selection of downstream pathways.^67^ ^,^ ^68^ In addition, regulatory factors such as post-translational modifications and isoforms generate tissue-specific responses to the same stimulus.^57^ ^,^ ^58^ When these multilayered controls are considered, the seemingly paradoxical phenomena of aging acceleration driven by elevated basal p53 activity and tumor clonal dominance driven by p53 functional attenuation during tumor progression can be coherently integrated as differences in temporal context, cellular environment, and selective pressures.^33^ ^,^ ^34^ ^,^ ^69^

In summary, p53 exhibits AP-like duality, contributing to tumor suppression and reproductive adaptation in early life, while dysregulation of its activity with aging promotes accelerated aging and cancer development. Viewing p53 within a multidimensional context that includes its interactions with mTOR/AMPK/autophagy, cellular senescence, and SASP provides a clearer framework for understanding the age-dependent pathogenesis of endometrial cancer and reproduction-related disorders.

#### 3.3.3. Beyond PI3K and p53: integration of the tumor microenvironment

In the present study, the PI3K and p53 pathways are positioned as central regulatory axes; however, these pathways alone are insufficient to fully account for the complexity of tumor biology. In recent years, the concept of antagonistic pleiotropy (AP) has evolved beyond its original focus on the age-dependent effects of single genes and has been expanded into a multidimensional trade-off theory applicable to complex diseases, including cancer. AP is now recognized as an integrative framework encompassing the tumor immune microenvironment, stromal interactions, epigenetic regulation, and metabolic inflammation. While these processes contribute to tissue homeostasis under physiological conditions, they may become tumor-promoting under chronic stress or aging. Therefore, endometrial cancer should be understood as a breakdown of multilayered networks driven by evolutionary trade-offs.

First, the tumor immune microenvironment functions as an antitumor defense system that eliminates malignant cells. During the early stages of tumorigenesis, immune surveillance contributes to tumor control; however, persistent antigenic stimulation and metabolic stress induce T cell exhaustion, thereby facilitating immune evasion.^70^ This transition from immune surveillance to immunosuppression represents a prototypical example of AP. Indeed, tumor metabolism has been reported to establish an immunosuppressive microenvironment, inhibiting effector T cells while promoting the expansion of regulatory T cells.^71^ Furthermore, sustained antigenic stimulation stabilizes T cell exhaustion through epigenetic reprogramming.^72^ Consistent with these findings, immune evasion mechanisms have also been documented in endometrial cancer.^73^

Next, the tumor stroma is indispensable for understanding AP. Cancer-associated fibroblasts (CAFs) and immune cells normally contribute to tissue repair and homeostasis^74^ ^,^ ^75^; however, under conditions of chronic inflammation, they undergo reprogramming and promote tumor proliferation, angiogenesis, invasion, and metastasis.^76^ This phenomenon represents a classical example of AP, often described as the contrast between wound healing and cancer progression. Moreover, tumor progression is governed by the dynamic interactions among tumor cells, immune cells, and stromal components.

Furthermore, epigenetic regulation is essential for development and cellular differentiation but, in cancer, induces immunosuppression, enhanced cellular plasticity, and therapeutic resistance.^77^ Epigenetic regulators are evolutionarily conserved, with some undergoing positive selection, suggesting that adaptive evolution may increase disease susceptibility.^78^ Thus, this mechanism exemplifies AP through the trade-off between the benefits of cellular plasticity and the promotion of tumor evolution.

In addition, metabolic adaptation supports cell survival under hypoxia and nutrient deprivation; however, within the tumor microenvironment, hypoxia, lactate accumulation, and chronic inflammation induce immunosuppression and promote tumor progression.^79^ ^,^ ^80^ Such metabolic reprogramming facilitates immune evasion and constitutes another example of AP. Moreover, metabolism, epigenetic regulation, and immune responses form an interconnected network that underlies tumor adaptation and evolution.^70^ Therefore, immunometabolism provides a critical theoretical foundation for understanding evolutionary trade-offs in tumorigenesis.

### 3.4. Endometrial cancer as an evolutionary trade-off

#### 3.4.1. Endometrial cancer from the perspective of antagonistic pleiotropy

The theory of antagonistic pleiotropy (AP) describes an evolutionary trade-off in which traits that enhance survival or reproductive success early in life become detrimental later in life, providing an important framework for understanding aging, cancer, and chronic diseases.^44^ ^,^ ^46^ ^,^ ^50-53^ ^,^ ^81^ ^,^ ^82^ Genetic variants that optimize growth and reproduction in early life have been shown to increase the risk of age-related diseases, including cancer, with aging.^81^

Applied to endometrial cancer, features that support fertility during reproductive years—such as high regenerative capacity, estrogen responsiveness, and proliferative potential—can be interpreted as early-life benefits that later incur costs. With aging, these traits interact with declining DNA repair capacity, genomic instability, hormonal changes, chronic inflammation, and cellular senescence, shifting toward abnormal proliferation and increased cancer risk (Figure 2, lower left, lower right, and upper right). Age-associated dysregulation of epigenetic factors or non-coding RNAs that are advantageous for tissue repair early in life may further promote tumorigenesis in the endometrium, which undergoes repeated cycles of regeneration.^83^

Tumor suppressors such as p53 exemplify AP: they protect against cancer early in life, whereas excessive activation with aging can impair tissue repair through senescence and stem cell dysfunction, and reduced p53 function may be selectively favored during carcinogenesis.^55^ ^,^ ^84^ Likewise, PI3K–AMPK–mTOR signaling is essential for reproductive adaptation and endometrial homeostasis, but age-related hyperactivation or dysregulation promotes aging, endometrial dysfunction, and tumorigenesis.^25^ Cellular senescence itself represents a canonical AP mechanism, acting as a tumor suppressor early in life but contributing to chronic inflammation and cancer through SASP accumulation in old age.^53^

Although endometrial cancer is multifactorial and cannot be explained solely by AP effects of individual genes, and empirical evidence for AP in humans remains limited,^81^ this framework provides an evolutionarily informed explanation for why tumors of reproductive and hormone-dependent tissues preferentially arise in middle to late adulthood.

#### 3.4.2. Endometrial cancer from the perspective of evolutionary mismatch

Throughout most of human evolution, food availability was unstable, and adaptive mechanisms centered on the AMPK–mTOR axis evolved to efficiently store energy and allocate it to tissue regeneration and reproduction. These nutrient- and energy-sensing systems are evolutionarily ancient, with core components of AMPK and mTOR emerging before the last eukaryotic common ancestor.^85^ In females, traits such as cyclic endometrial regeneration, efficient energy storage, metabolic flexibility, and hormone responsiveness evolved to support pregnancy and reproduction through interconnected estrogen/progesterone,^86^ PI3K–AKT–mTOR,^87^ Wnt/TGF-*β,* ^88^ ^,^ ^89^ and AMPK–mTOR signaling networks.^90^

In modern societies, persistent overnutrition, obesity, insulin resistance, physical inactivity, chronic inflammation, and excessive estrogen exposure represent a prototypical evolutionary mismatch, in which adaptations once beneficial for survival and reproduction now promote disease. This concept is exemplified by the “thrifty” phenotype hypothesis, whereby metabolic adaptations to early-life undernutrition increase disease risk in nutrient-rich adult environments.^91^

This mismatch is particularly relevant to Type I endometrial cancer, in which hormone dependence and dysregulated metabolism are central to carcinogenesis. Overnutrition, obesity, chronic insulin/IGF-1 signaling, estrogen excess, and obesity-associated inflammation continuously stimulate the endometrium, driving excessive proliferation, metabolic and epigenetic alterations, endometrial hyperplasia, and the accumulation of driver mutations.^92^ In addition, increased lifespan, population aging, reduced parity, and prolonged cumulative hormone exposure further unmask cancer risks that were less apparent in ancestral environments. Accordingly, lifestyle modification and metabolic regulation—such as weight control, balanced diet, physical activity, and hormone management—can be framed as preventive strategies that mitigate evolutionary mismatch, with chronic PI3K–AKT–mTOR hyperactivation representing a central molecular link.

By contrast, p53 dysregulation is better understood not as a direct adaptive response to modern environments but as a consequence of chronic stress and aging during tumor progression. Sustained inflammation, oxidative stress, and DNA damage alter p53 response modes, promoting cellular senescence and tissue decline, and over time favor the selection of clones with reduced p53 function. This two-step process—chronic stress–induced regulatory disruption followed by selection of p53-deficient clones—indirectly positions p53 dysregulation within the framework of evolutionary mismatch.

#### 3.4.3. Endometrial cancer from the perspective of life history theory (LHT)

From the perspective of evolutionary Life History Theory (LHT), traits such as timing of reproduction, parity, number of ovulatory cycles, age at menopause, lifespan, and adipose energy storage have been shaped to maximize reproductive success but may involve trade-offs that increase postmenopausal endometrial burden and cancer risk.^93^ Consistent with this framework, epidemiological studies have shown that early menarche, late menopause, low parity, and nulliparity are associated with increased endometrial cancer risk, emphasizing the importance of cumulative lifetime estrogen exposure.^94^ ^,^ ^95^ Obesity, overnutrition, insulin resistance, and chronic inflammation are also strongly linked to endometrial cancer risk,^96^ ^,^ ^97^ supporting molecular models in which adipose-derived estrogen, insulin/IGF-1 signaling, and inflammation provide sustained proliferative stimuli to the endometrium.^98^ Collectively, these findings support a pathway in which overnutrition leads to obesity, hyperinsulinemia and elevated estrogen, chronic inflammation, persistent endometrial stimulation, and ultimately carcinogenesis.

Comparative and theoretical studies further suggest trade-offs between reproductive investment and cancer susceptibility, providing a conceptual basis for linking reproductive strategies and life-history traits to tumor risk.^99-101^ However, although these associations are broadly consistent with epidemiological and molecular evidence, direct empirical tests applying LHT or antagonistic pleiotropy to endometrial cancer remain limited.

In conclusion, evolutionary mismatch primarily explains how modern metabolic environments impose excessive proliferative pressure on the endometrium, whereas LHT emphasizes intrinsic trade-offs between reproductive investment and somatic maintenance. In endometrial cancer, metabolic dysregulation and chronic PI3K–AKT–mTOR activation are more readily interpreted through evolutionary mismatch, while estrogen exposure, reproductive history, and age-related risk align more naturally with Life History Theory. Together, these frameworks offer complementary perspectives on the evolutionary basis of endometrial carcinogenesis.

#### 3.4.4. Testable hypotheses and predictive implications

Estrogen is a principal hormone that promotes endometrial proliferation, and its effects are mediated through activation of the PI3K–AKT–mTOR signaling pathway. The PI3K pathway regulates nutrient sensing and cellular growth and is indispensable for development and reproductive function; however, its sustained activation has been shown to promote cellular senescence and disease onset.^102^ According to the theory of antagonistic pleiotropy (AP), such signaling pathways that contribute to development and reproduction support endometrial function and fertility during early life but may exert deleterious effects under conditions of aging or metabolic dysregulation, leading to cellular senescence and functional decline.^41-45^ Accordingly, changes in the reproductive patterns of modern women are understood as an evolutionary mismatch from ancestral environments and are associated with an increased risk of estrogen-dependent diseases.^94^

In particular, constitutive activation of the PI3K pathway due to PTEN mutations is deeply involved in the development of endometrial hyperplasia and endometrial carcinoma.^12^ ^,^ ^13^ ^,^ ^18^ ^,^ ^19^ ^,^ ^38^ ^,^ ^39^ Furthermore, the mTOR pathway constitutes part of an evolutionarily conserved network governing growth and stress responses, aligning with the AP framework in which early adaptive advantages are offset by increased risks of infertility later in life.^103^ Similarly, the tumor suppressor p53 contributes to survival during the reproductive period by eliminating abnormal cells; however, excessive activation may impair stem cell function and tissue regeneration, thereby contributing to age-related pathologies.^46^

Taken together, prolonged estrogen exposure associated with modern reproductive patterns may, in the context of evolutionary mismatch, lead to persistent modulation of PI3K–AKT–mTOR and p53 signaling, thereby providing a molecular basis for the development of endometrial hyperplasia and tumorigenesis. These findings suggest that evolutionary mismatch and antagonistic pleiotropy offer an integrated conceptual framework for explaining the accumulation of molecular abnormalities in endometrial cancer. Nevertheless, these conclusions derived from evolutionary medicine remain largely theoretical and interpretive and have yet to be systematically and rigorously validated in clinical settings.

## 4. Discussion

Molecular classification by TCGA has clarified that the pathogenesis of endometrial cancer is not driven by a single oncogenic event but instead reflects a complex network in which defects in DNA repair, constitutive activation of PI3K–AKT–mTOR signaling, and disruption of the p53 pathway interact with one another.^6^ This review proposes that these abnormalities should not be viewed merely as the stochastic accumulation of mutations, but rather as evolutionary by-products of biological systems that were originally optimized for reproduction.

First, the PI3K–AKT–mTOR pathway represents an evolutionarily highly conserved nutrient-sensing system that plays fundamental roles in reproduction during early life, including follicular development, endometrial proliferation, decidualization, embryo receptivity, and placental formation.^38^ ^,^ ^39^ However, modern environmental factors such as obesity, overnutrition, and insulin resistance chronically stimulate this pathway, leading to excessive endometrial proliferation, synergistic effects with estrogen overexposure, and aberrant activation of the stem cell niche.^41-43^ These phenomena exemplify the antagonistic pleiotropy (AP) paradigm, in which early-life benefits (promotion of reproduction) are converted into late-life costs (tumorigenesis). In contrast, the p53 pathway is essential for genome protection and tumor suppression and plays critical roles during the reproductive period in endometrial decidualization, placental development, and stress regulation in placental trophoblasts. Nevertheless, p53 activity must be exquisitely regulated in reproduction: excessive activation can lead to miscarriage or implantation failure, whereas insufficient activity may increase the risk of preterm birth.^59-63^ Aging, inflammation, oxidative stress, and epigenetic alterations can disrupt the homeostasis of p53 regulatory systems, potentially driving tumor phenotypes exemplified by the copy-number–high (CNH) subtype. This represents a canonical AP scenario in which the early-life benefit of genomic protection is transformed into late-life costs in the form of tissue dysfunction and tumor promotion. Moreover, these two pathways intersect through senescent cells, SASP, the stem cell niche, and an inflammatory microenvironment, thereby mutually reinforcing phenotypes such as TP53 mutation and PI3K hyperactivation. Evolutionary mismatch factors—including obesity, dysregulated glucose metabolism, chronic inflammation, delayed childbearing, and increased lifetime menstrual cycles—may further accelerate this reinforcing loop, contributing in particular to the accumulation of PI3K–AKT–mTOR pathway alterations in type I tumors and disruption of the p53 pathway in type II tumors.

This review has several limitations. First, although the application of evolutionary mismatch and AP theory to endometrial cancer is conceptually coherent, empirical evidence remains limited. In particular, while experimental studies in animal models and cell systems suggest causal links whereby pathways advantageous in early life (such as PI3K–AKT–mTOR and p53) contribute to tumor promotion in later life, longitudinal human data are scarce. Second, the risk architecture of endometrial cancer is shaped by complex interactions among metabolic status, lifestyle factors, reproductive history, hormonal milieu, and genetic background, and it is difficult to fully explain the directionality and interplay of these factors within a single evolutionary medicine framework. Third, although understanding of molecular pathogenesis based on TCGA classification has advanced rapidly, gaps remain between these insights and routine clinical practice, as well as limitations in generalizability due to underrepresentation of diverse populations, including Asian cohorts. Accordingly, the conceptual framework proposed in this review should be regarded as hypothesis-generating at present.

In summary, the molecular abnormalities observed in endometrial cancer—PI3K pathway dysregulation, p53 pathway disruption, and DNA repair defects—can be interpreted not simply as random neoplastic events, but as maladaptive manifestations of systems that evolved to optimize reproductive fitness under ancestral conditions. The frameworks of antagonistic pleiotropy and evolutionary mismatch offer a powerful theoretical basis for understanding endometrial carcinogenesis as part of a continuous spectrum linking reproduction, aging, and cancer.

## 5. Future perspectives

An evolutionary medicine perspective has the potential to deepen our understanding of endometrial cancer pathophysiology and to inform preventive strategies; however, several challenges remain to be addressed. First, longitudinal studies are required to link the antagonistic pleiotropic (AP) duality of PI3K–AKT–mTOR and p53 signaling—pathways that govern growth, metabolism, and aging—with life-long reproductive strategies and metabolic environments. In particular, it is essential to elucidate how changes in hormonal milieu, reproductive history, body fat composition, and insulin resistance from puberty through postmenopause are associated with subtype-specific risks of endometrial cancer. Second, from the perspective of evolutionary mismatch, multilevel studies are needed to evaluate the extent to which modern lifestyle factors—such as high-calorie diets, sedentary behavior, low physical activity, and declining parity—chronically elevate PI3K–AKT–mTOR activity and thereby contribute to accelerated aging and tumorigenesis. Third, cross-disciplinary investigations into shared dysregulation of the mTOR–p53–senescence–inflammation axis in conditions such as preterm birth, miscarriage, placental dysfunction, and endometrial cancer may enable the development of novel disease models that integrate reproductive medicine and tumor biology. These insights may also translate directly into preventive medicine through obesity management, metabolic optimization, and hormonal balance in women of reproductive age.

## 6. Conclusion

In this review, we have reexamined the pathogenesis of endometrial cancer by integrating TCGA molecular classification, the classical dualistic model, and dysregulation of the PI3K–AKT–mTOR and p53 pathways within the frameworks of evolutionary mismatch, life history theory (LHT), and antagonistic pleiotropy (AP). These pathways contribute to reproductive success, tissue regeneration, and metabolic homeostasis in early life, yet may be redirected toward accelerated aging, chronic inflammation, and tumorigenesis under modern environmental exposures and age-related loss of homeostasis. This trade-off between early-life benefits and late-life costs provides a unifying perspective for understanding endometrial carcinogenesis at the intersection of reproductive biology, tumor biology, and evolutionary medicine. Overall, metabolic and endocrine mismatches in contemporary environments likely drive excessive activation of growth, reproductive, and repair pathways that have been maintained through AP, thereby contributing to earlier onset and increasing incidence of endometrial cancer.

## Data Availability

This is a review article and does not contain any new data.
